# Risk stratification system and web-based nomogram constructed for predicting the overall survival of primary osteosarcoma patients after surgical resection

**DOI:** 10.3389/fpubh.2022.949500

**Published:** 2022-08-05

**Authors:** Bing Gao, Meng-die Wang, Yanan Li, Fei Huang

**Affiliations:** ^1^Department of Orthopedics, China-Japan Union Hospital of Jilin University, Changchun, China; ^2^Department of Neurology, China-Japan Union Hospital of Jilin University, Changchun, China; ^3^Department of Pediatrics, The First Hospital of Jilin University, Changchun, China

**Keywords:** osteosarcoma, surgical resection, overall survival, nomogram, risk stratification, web application

## Abstract

**Background:**

Previous prediction models of osteosarcoma have not focused on survival in patients undergoing surgery, nor have they distinguished and compared prognostic differences among amputation, radical and local resection. This study aimed to establish and validate the first reliable prognostic nomogram to accurately predict overall survival (OS) after surgical resection in patients with osteosarcoma. On this basis, we constructed a risk stratification system and a web-based nomogram.

**Methods:**

We enrolled all patients with primary osteosarcoma who underwent surgery between 2004 and 2015 in the Surveillance, Epidemiology, and End Results (SEER) database. In patients with primary osteosarcoma after surgical resection, univariate and multivariate cox proportional hazards regression analyses were utilized to identify independent prognostic factors and construct a novel nomogram for the 1-, 3-, and 5-year OS. Then the nomogram's predictive performance and clinical utility were evaluated by the concordance index (C-index), receiver operating characteristic (ROC) curves, calibration curves, and decision curve analysis (DCA).

**Result:**

This study recruited 1,396 patients in all, with 837 serving as the training set (60%) and 559 as the validation set (40%). After COX regression analysis, we identified seven independent prognostic factors to develop the nomogram, including age, primary site, histological type, disease stage, AJCC stage, tumor size, and surgical method. The C-index indicated that this nomogram is considerably more accurate than the AJCC stage in predicting OS [Training set (HR: 0.741, 95% CI: 0.726–0.755) vs. (HR: 0.632, 95% CI: 0.619–0.645); Validation set (HR: 0.735, 95% CI: 0.718–0.753) vs. (HR: 0.635, 95% CI: 0.619–0.652)]. Moreover, the area under ROC curves, the calibration curves, and DCA demonstrated that this nomogram was significantly superior to the AJCC stage, with better predictive performance and more net clinical benefits.

**Conclusion:**

This study highlighted that radical surgery was the first choice for patients with primary osteosarcoma since it provided the best survival prognosis. We have established and validated a novel nomogram that could objectively predict the overall survival of patients with primary osteosarcoma after surgical resection. Furthermore, a risk stratification system and a web-based nomogram could be applied in clinical practice to assist in therapeutic decision-making.

## Introduction

Osteosarcoma has a population incidence of only 3/million/year, with a male-to-female ratio of 1.4:1, and most commonly affects children and adolescents. In the United States, osteosarcoma affects 2% of children (1 to 14 years) with cancer and 3% of adolescents (15 to 19 years) (1–3). The pathogenesis of osteosarcoma is related to the rapid proliferation of bone, which is manifested in the growth spurt during puberty. It is more common in the metaphysis of long bones of the extremities, such as the proximal tibia and distal femur (4, 5). Axial skeletal osteosarcoma involvement occurs mainly in elderly patients, accounting for approximately 10%, most cases occurring in the pelvis (2, 6).

With surgical advancements in recent decades, limb salvage surgery has gradually replaced amputation as the principal surgical modality for safely removing malignancies (2). Salvage surgery could preserve as much function as feasible to achieve microscopically clear surgical margins (7). Compared with surgery alone, surgical resection combined with multimodal chemotherapy can enhance disease-free survival in osteosarcoma from 10–20 to 60–70% and is recognized as the most effective treatment (2, 4, 8, 9). Salvage surgery has been more advanced in recent years thanks to advancements in adjuvant chemotherapy research, high-intensity focused ultrasound ablation (HIFU), and computer-aided navigation systems (CANS) (10). However, although both local resection and radical resection are considered salvage surgeries, previous studies have failed to distinguish between the two or develop an effective postoperative predictive model for osteosarcoma patients (11–16).

As the gold standard for predicting the prognosis of malignant tumors, the American Joint Committee on Cancer (AJCC) staging system (i.e., the TMN staging system) is widely recognized, yet it still has limitations (17, 18) The AJCC staging system neglects individual characteristics such as age, sex, histology type, etc., making it insufficient to predict the individualized probability of survival after surgical resection in patients with osteosarcoma. Nomograms, which graphically and intuitively depict statistical prediction models, have been extensively applied to study the prognosis of cancer patients (17). Compared with the AJCC staging system, the nomogram integrates cancer patients' clinical characteristics and tumor status, allowing for a more accurate assessment of individual survival and compensating for the AJCC system's inadequacies (17, 19).

Previous prognostic nomograms for osteosarcoma have reported that age, sex, tumor size, primary site, grade, disease stage, AJCC stage, surgery, chemotherapy, radiotherapy, distant metastasis, and so on were associated with prognostic survival (11–16, 20–22). Still, they have not yet explicitly focused on prognostic risk factors in postoperative patients, nor have they distinguished and compared survival differences among patients after amputation, radical resection, and local resection. This study established and validated the first comprehensive and practical nomogram for predicting overall survival (OS) after surgical resection in patients with osteosarcoma based on the SEER database. This nomogram enabled orthopedic surgeons to efficiently formulate surgical strategies in the perioperative period and strengthen their prospective decision-making capacity.

## Materials and methods

### Data source and variable definitions

The Surveillance, Epidemiology, and End Results (SEER) database is a valuable data source for studying the incidence and survival of rare cancer populations in the United States. The SEER database collects information on approximately 450,000 malignant and *in situ* carcinomas cases annually, including patient tumor characteristics, stage at diagnosis, and mortality outcomes (23).

We recruited all cases required for this study from the SEER database (https://seer.cancer.gov). Inclusion criteria were as follows: (1) The morphologic code was bone and joint (ICD-O3, i.e., International Classification of Disease for Oncology-3rd edition); (2) The pathological typing code was osteosarcoma (9,180–9,187, 9,192–9,194); (3) Diagnosed by positive histological evidence; (4) Diagnosed between 2004 and 2015; (5) The primary site of the tumor was limbs or pelvis (400–403,414); (6) Surgery performed. Exclusion criteria were as follows:(1) Demographic variables (age, sex, race) were unavailable; (2) Tumor characteristics (tumor size, laterality, grade, SEER stage, AJCC stage) were unavailable; (3) Survival months <1 month.

### Variable definitions

The following demographic and clinical information were gathered for this study: age (<18,18–47,>47), race (black, white, other), primary site (limbs, pelvis), histological type (peripheral, central, NOS), laterality (left, right, unpaired site), disease stage (distant, localized, regional), AJCC stage (I–IV), grade (I–IV), tumor size (<70, 70–139, >139), surgery (local resection, radical resection, amputation), sex, year of diagnosis, radiotherapy, and chemotherapy.

Because age and tumor size were continuous variables, we applied the X-tile procedure to determine optimal cut-offs, defining age groups (18, 18–47, >47) and tumor size groups (70, 70–139, >139) (24). Due to the small sample size of some subtypes, we integrated all histological types of osteosarcomas by the ICD-O-3 codes. “Central osteosarcoma” comprised 9,181/3, 9,182/3, 9,183/3, 9,185/3, 9,186/3; 9,187/3, “Peripheral osteosarcoma” included 9,192/3, 9,193/3, 9,194/3; “Osteosarcoma, NOS” represented for 9,180/3, i.e., osteosarcoma not otherwise specified (25). For “Grade,” “Grade I” meant good differentiation, “Grade II” meant moderate differentiation, “Grade III” meant poor differentiation, and “Grade IV” meant undifferentiated. For “Disease stage,” “Localized” denoted tumor confined to the periosteum, “Regional” denoted adjacent tissues or lymph node involvement, and “Distant” denoted distal site metastasis (26, 27).

For “Primary site,” primary tumor sites were combined as “Limbs” and “Pelvis.” “Limbs” comprised “C40.0:Long bones of the upper limb”, “C40.1:Short bones of the upper limb”, “C40.2:Long bones of the lower limb”, and “C40.3:Short bones of the lower limb”; Pelvis represented “C41.4-Pelvic bones” (4, 6, 28). This study categorized surgical methods for patients with osteosarcoma into local resection, radical resection, and amputation. “Local resection” comprised surgery codes: 15,19,25,26, “Amputation” included: 40–42, 50–54, and “Radical resection” represented “surgery code=30”. To study the prognosis and survival of patients, we determined overall survival (OS) as the primary endpoint.

### Statistical analysis

We used R (version 4.1.2; http://www.r-project.org) for all analyses, with “survival,” “rms,” “nomogramFormula,” “ggplot2,” and other R packages. All statistical tests were two-sided, and statistical significance was defined as a *P* < 0.05. Patients from the training set were used to develop the nomogram and risk stratification system, which was subsequently verified with the validation set. The univariate analysis was used to exclude factors unrelated to the postoperative OS in patients with osteosarcoma, followed by multivariate analysis to identify independent prognostic factors. After that, we selected the above statistically significant independent factors to establish the nomogram (29).

The concordance index (C-index) and the area under the curve (AUC) were calculated to evaluate the nomogram's discrimination. Then, the calibration curve was utilized to verify the accuracy of the nomogram. Furthermore, to assess clinical utility, decision curve analysis (DCA) was devised to compare the clinical net benefit of the nomogram with the AJCC stage (30).

In addition, we utilized the X-Tile software to figure out the optimal cut-off values for each patient's nomogram total score (24, 31). Based on these values, we divided the patients into three groups to establish a risk stratification system: low-risk, medium-risk, and high-risk. The Kaplan-Meier curve and log-rank test verified differences in OS of patients in each risk group. Moreover, the package of “DynNom” was applied to create a web-based nomogram that allows orthopedic surgeons immediately and accurately assess postoperative OS in patients with osteosarcoma.

## Results

### Patient characteristics

**Figure 1:**
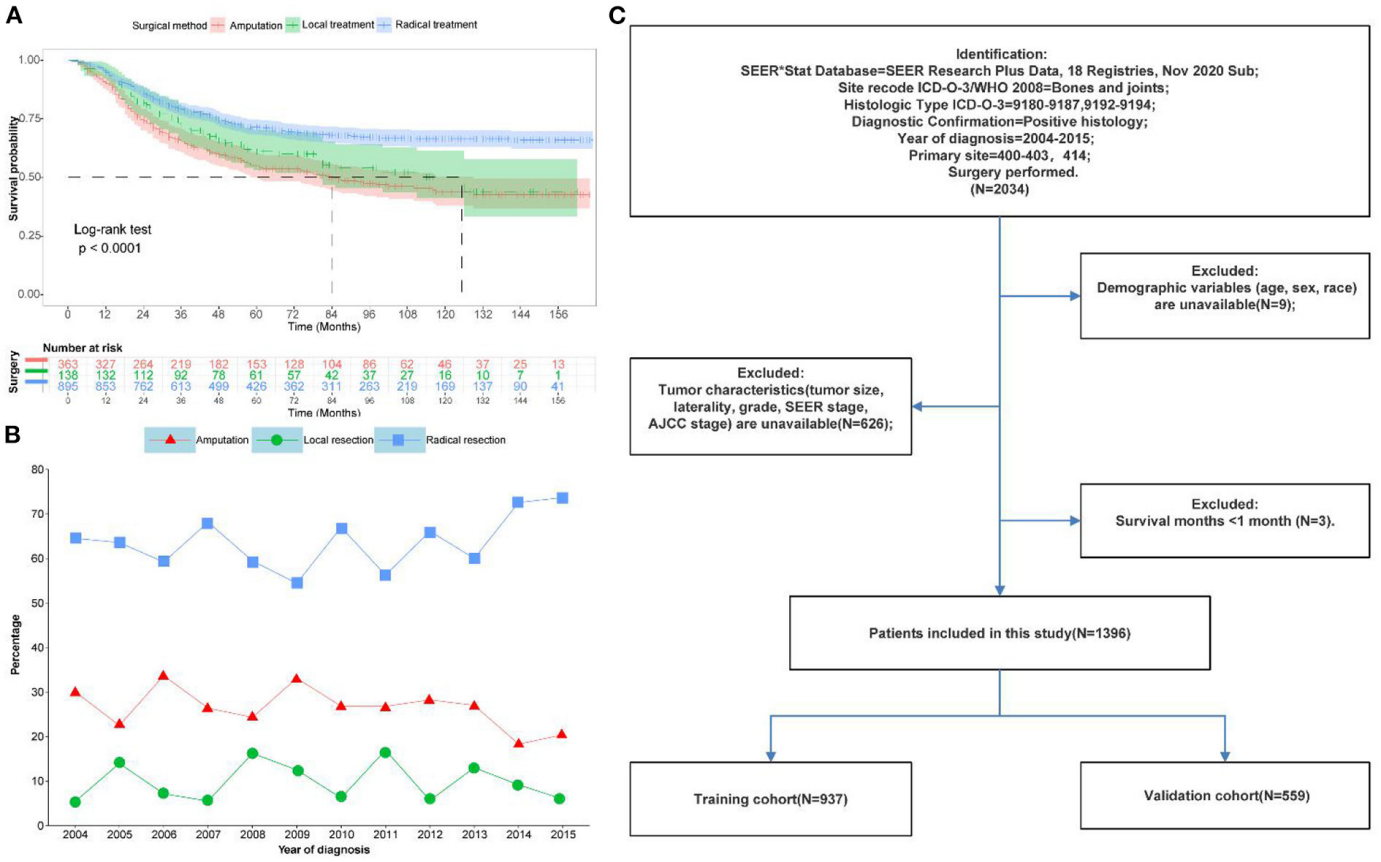
Data of patients with primary osteosarcoma after surgical resection in this study. **(A)** Kaplan-Meier survival curves for different surgical options. **(B)** Trends in different surgical options from 2004 to 2015 **(C)** Flow chart of patient selection.

This study recruited 1,396 patients with primary osteosarcoma who underwent surgery from the SEER database between 2004 and 2015. Figure 1C depicted a flowchart of the patient selection process. Patients were randomly divided into a training set and a validation set with a ratio of 6:4. The training set (*N* = 837,60%) was used for prognostic model building and internal validation, while the validation set (*N* = 559,40%) was for external validation. Figures 1A,B depicted the trends and KM survival curves of all patients who underwent different surgical methods between 2004 and 2015. Radical surgery was the first choice for patients with primary osteosarcoma since it provided the best prognosis for survival.

Supplementary Figure S1 illustrated selecting the best cut-off values for age and tumor size. The median age of all patients was 17 years (Table 1), with cut-off values of 18 and 47 years (Supplementary Figures S1A,B). The median tumor size was 94.5 mm (Table 1), with cut-off values of 70 and 139 mm (Supplementary Figures S1C,D). We calculated the median follow-up time of 88 months (95% CI: 83–93 months) for all patients by the reverse Kaplan-Meier method. Of the recruited cases, 658 patients were diagnosed from 2004 to 2009, 738 patients were diagnosed from 2010 to 2014, with 772 men and 624 women, and most were white (1,048, 75.1%). Table 1 summarized the demographic and clinical characteristics of all selected patients. The most common tumor primary site was the limbs (1,316, 94.3%), while the pelvic (80, 5.7%) was relatively rare. The histological type could not be determined in most patients (858, 61.5%), with the central type (414, 29.7%) more common than the peripheral type (124, 8.9%). The risk of catching primary osteosarcoma on the left (706, 50.6%) and right (670, 48.0%) sides was nearly comparable. As for tumor grade, there were significantly more patients with high-grade (grade III/IV, 89.2% in total) than low-grade (grade I/II, 10.8% in total). According to the disease stage, regional (671, 48.1%) accounted for nearly half of the cohort, followed by localized (479, 34.3%) and distant (246,17.6%). According to the AJCC stage, most patients (72.3%) were in Stage II, 10.5% in Stage I, 15.5% in Stage IV, and 1.6% in Stage III. The majority of patients received chemotherapy (1,208, 86.5%), but most refused radiotherapy (1,356, 97.1%). In terms of surgical options, radical resection was the first choice (895, 64.1%), 363 (26.0%) cases underwent amputation, and 138 (9.9%) opted for local excision. There was no statistically significant difference between training and validation sets (*P* > 0.05).

**Table 1:** Baseline demographic and clinicopathological characteristics of the patients.

**Characteristics**	**Total** ***N* = 1,396, *N* (%)**	**Training** ***N* = 837, *N* (%)**	**Validation** ***N* = 559, *N* (%)**	***P*-value**
**Age (years)**				0.698
Mean (SD)	24.6 (17.5)	24.5 (17.3)	24.8 (17.9)	
Median [Min, Max]	17.0 [3.00, 88.0]	17.0 [3.00, 88.0]	17.0 [3.00, 87.0]	
**Age (years)**				0.988
<18	705 (50.5%)	424 (50.7%)	281 (50.3%)	
>47	179 (12.8%)	107 (12.8%)	72 (12.9%)	
18–47	512 (36.7%)	306 (36.6%)	206 (36.9%)	
**Year of diagnosis**				0.101
2004–2009	658 (47.1%)	410 (49.0%)	248 (44.4%)	
2010–2015	738 (52.9%)	427 (51.0%)	311 (55.6%)	
**Sex**				0.138
Female	624 (44.7%)	388 (46.4%)	236 (42.2%)	
Male	772 (55.3%)	449 (53.6%)	323 (57.8%)	
**Race**				0.699
Black	218 (15.6%)	134 (16.0%)	84 (15.0%)	
Others	130 (9.3%)	74 (8.8%)	56 (10.0%)	
White	1,048 (75.1%)	629 (75.1%)	419 (75.0%)	
**Primary site**				1
Limbs	1,316 (94.3%)	789 (94.3%)	527 (94.3%)	
Pelvic	80 (5.7%)	48 (5.7%)	32 (5.7%)	
**Histological type**				0.506
Central osteosarcoma	414 (29.7%)	258 (30.8%)	156 (27.9%)	
Osteosarcoma, NOS	858 (61.5%)	506 (60.5%)	352 (63.0%)	
Peripheral osteosarcoma	124 (8.9%)	73 (8.7%)	51 (9.1%)	
**Laterality**				0.84
Left	706 (50.6%)	421 (50.3%)	285 (51.0%)	
Right	670 (48.0%)	405 (48.4%)	265 (47.4%)	
Unpaired site	20 (1.4%)	11 (1.3%)	9 (1.6%)	
**Grade**				0.756
Grade I	57 (4.1%)	32 (3.8%)	25 (4.5%)	
Grade II	94 (6.7%)	59 (7.0%)	35 (6.3%)	
Grade III	417 (29.9%)	244 (29.2%)	173 (30.9%)	
Grade IV	828 (59.3%)	502 (60.0%)	326 (58.3%)	
**Disease stage**				0.485
Distant	246 (17.6%)	156 (18.6%)	90 (16.1%)	
Localized	479 (34.3%)	284 (33.9%)	195 (34.9%)	
Regional	671 (48.1%)	397 (47.4%)	274 (49.0%)	
**AJCC Stage**				0.328
Stage I	146 (10.5%)	87 (10.4%)	59 (10.6%)	
Stage II	1,010 (72.3%)	595 (71.1%)	415 (74.2%)	
Stage III	23 (1.6%)	17 (2.0%)	6 (1.1%)	
Stage IV	217 (15.5%)	138 (16.5%)	79 (14.1%)	
**Tumor size (mm)**				0.166
Mean (SD)	105 (57.9)	103 (56.9)	108 (59.3)	
Median [Min, Max]	94.5 [2.00, 750]	90.0 [2.00, 482]	100 [10.0, 750]	
**Tumor size (mm)**				0.373
<70	351 (25.1%)	216 (25.8%)	135 (24.2%)	
>139	294 (21.1%)	166 (19.8%)	128 (22.9%)	
70–139	751 (53.8%)	455 (54.4%)	296 (53.0%)	
**Surgical method**				0.411
Amputation	363 (26.0%)	225 (26.9%)	138 (24.7%)	
Local resection	138 (9.9%)	87 (10.4%)	51 (9.1%)	
Radical resection	895 (64.1%)	525 (62.7%)	370 (66.2%)	
**Radiation**				0.624
No/Unknown	1,356 (97.1%)	811 (96.9%)	545 (97.5%)	
Yes	40 (2.9%)	26 (3.1%)	14 (2.5%)	
**Chemotherapy**				0.576
No/Unknown	188 (13.5%)	109 (13.0%)	79 (14.1%)	
Yes	1,208 (86.5%)	728 (87.0%)	480 (85.9%)	

### Nomogram variable selection

Univariate Cox regression analysis revealed that eleven clinical variables were significantly associated with survival prognosis except for the year of diagnosis, race, and chemotherapy (Table 2). These significant variables included age, sex, primary site, histological type, laterality, grade, disease stage, AJCC stage, tumor size, radiation, and surgical method. These variables were incorporated into the multivariate cox analysis of OS. Age, primary site, histological type, disease stage, AJCC stage, tumor size, and surgical method were finally defined as independent prognostic factors for postoperative OS (Table 2).

**Table 2:** Univariate and multivariate analyses of overall survival (OS) in the training cohort.

**Characteristics**	**Univariate analysis**		**Multivariate analysis**	
	**HR (95%CI)**	**P**	**HR (95%CI)**	**P**
**Age (years)**
<18	**Reference**		**Reference**	
>47	3.114 (2.433–3.986)	**<0.001**	3.967 (2.897–5.432)	**<0.001**
18–47	1.329 (1.076–1.642)	**0.027**	1.946 (1.493–2.536)	**<0.001**
**Year of diagnosis**
2004–2009	**Reference**			
2010–2015	1.106 (0.912–1.343)	0.391		
**Sex**
Female	**Reference**		**Reference**	
Male	1.368 (1.132–1.654)	**0.007**	1.076 (0.846–1.368)	0.55
**Race**
Black	**Reference**			
Others	0.916 (0.634–1.323)	0.694		
White	0.787 (0.62–1)	0.1		
**Primary site**
Limbs	**Reference**		**Reference**	
Pelvic	2.887 (2.14–3.894)	**<0.001**	1.868 (1.208–2.888)	**0.005**
**Histological type**
Central osteosarcoma	**Reference**		**Reference**	
Osteosarcoma, NOS	1.189 (0.97–1.456)	0.161	1.282 (0.997–1.648)	0.053
Peripheral osteosarcoma	0.266 (0.145–0.487)	**<0.001**	0.406 (0.168–0.982)	**0.045**
**Laterality**
Left	**Reference**		**Reference**	
Right	1.046 (0.866–1.262)	0.697	1.051 (0.837–1.319)	0.669
Unpaired site	2.394 (1.267–4.522)	**0.024**	0.717 (0.302–1.703)	0.452
**Grade**
Grade I	**Reference**		**Reference**	
Grade II	1.645 (0.549–4.924)	0.456	1.193 (0.296–4.812)	0.804
Grade III	5.334 (2.036–13.978)	**0.004**	0.513 (0.071–3.72)	0.509
Grade IV	4.821 (1.852–12.554)	**0.007**	0.454 (0.063–3.277)	0.434
**Disease stage**
Distant	**Reference**		**Reference**	
Localized	0.202 (0.155–0.265)	**<0.001**	0.289 (0.120–0.699)	**0.006**
Regional	0.4 (0.324–0.494)	**<0.001**	0.461 (0.200–1.061)	0.069
**AJCC stage**
Stage I	**Reference**		**Reference**	
Stage II	3.685 (2.104–6.454)	**<0.001**	5.704 (1.218–26.71)	**0.027**
Stage III	8.58 (4.095–17.98)	**<0.001**	5.901 (0.913–38.13)	0.062
Stage IV	10.244 (5.757–18.229)	**<0.001**	6.987 (1.222–39.96)	**0.029**
**Tumor size (mm)**
<70	**Reference**		**Reference**	
>139	2.899 (2.187–3.842)	**<0.001**	1.774 (1.235–2.547)	**0.002**
70–139	1.593 (1.233–2.058)	0.003	1.329 (0.965–1.831)	0.082
**Surgical method**
Amputation	**Reference**		**Reference**	
Local resection	0.797 (0.586–1.085)	0.226	1.037 (0.703–1.529)	0.856
Radical resection	0.527 (0.431–0.645)	**<0.001**	0.762 (0.589–0.986)	**0.038**
**Radiation**
No/Unknown	**Reference**		**Reference**	
Yes	2.869 (1.923–4.281)	**<0.001**	1.635 (0.992–2.694)	0.054
**Chemotherapy**
No/Unknown	**Reference**			
Yes	1.158 (0.862–1.555)	0.415		

Bold values refer to P < 0.05 with statistical significance.

### Development and validation of the nomogram

**Figure 2:**
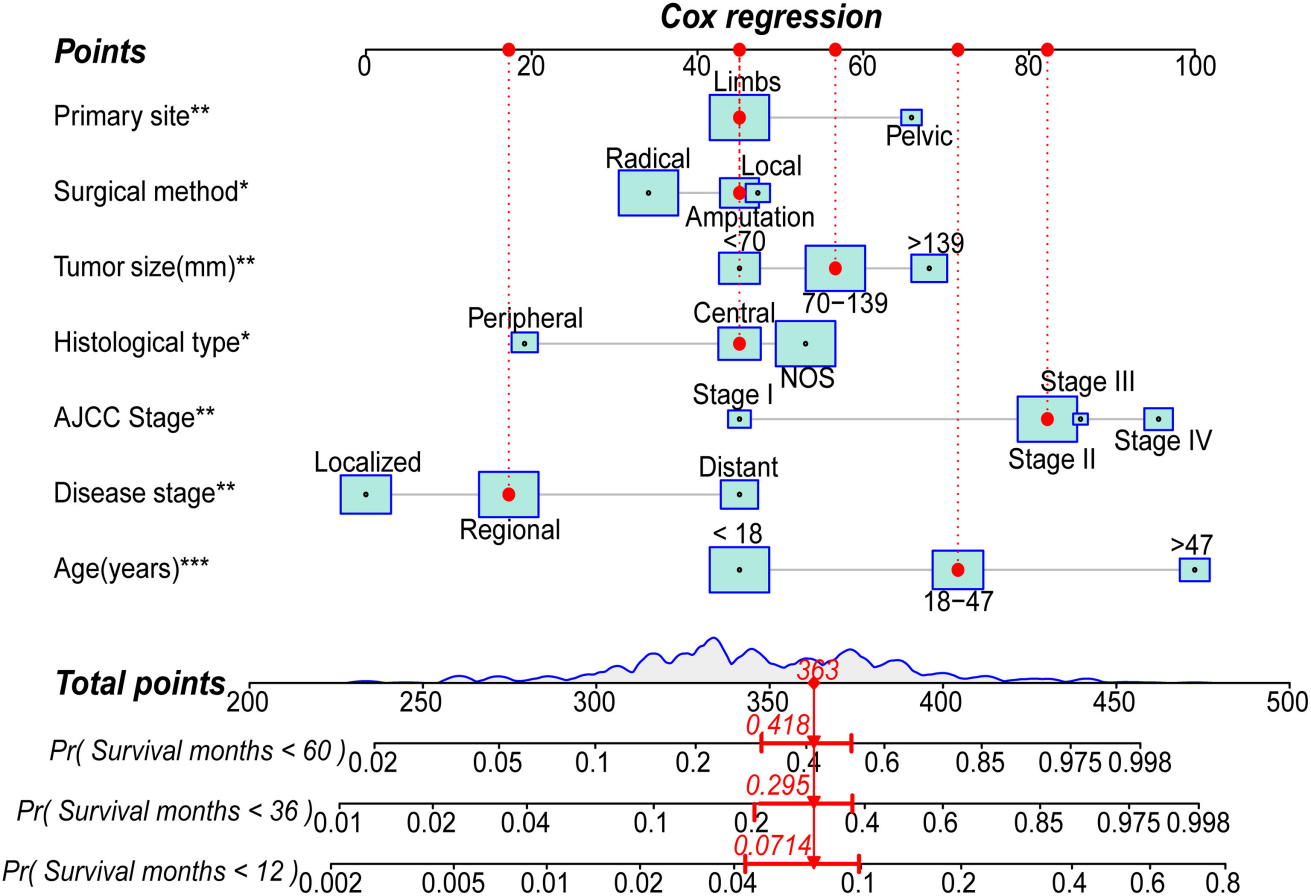
Nomogram for predicting the 1-,3- and 5-year overall survival (OS) rates of patients with primary osteosarcoma after surgical resection (*** *P* < 0.01; **P < 0.01; *P < 0.05; Radical, Radical resection; Local, Local resection; Central, Central osteosarcoma; Peripheral, Peripheral osteosarcoma; NOS, Osteosarcoma, NOS).

**Figure 3:**
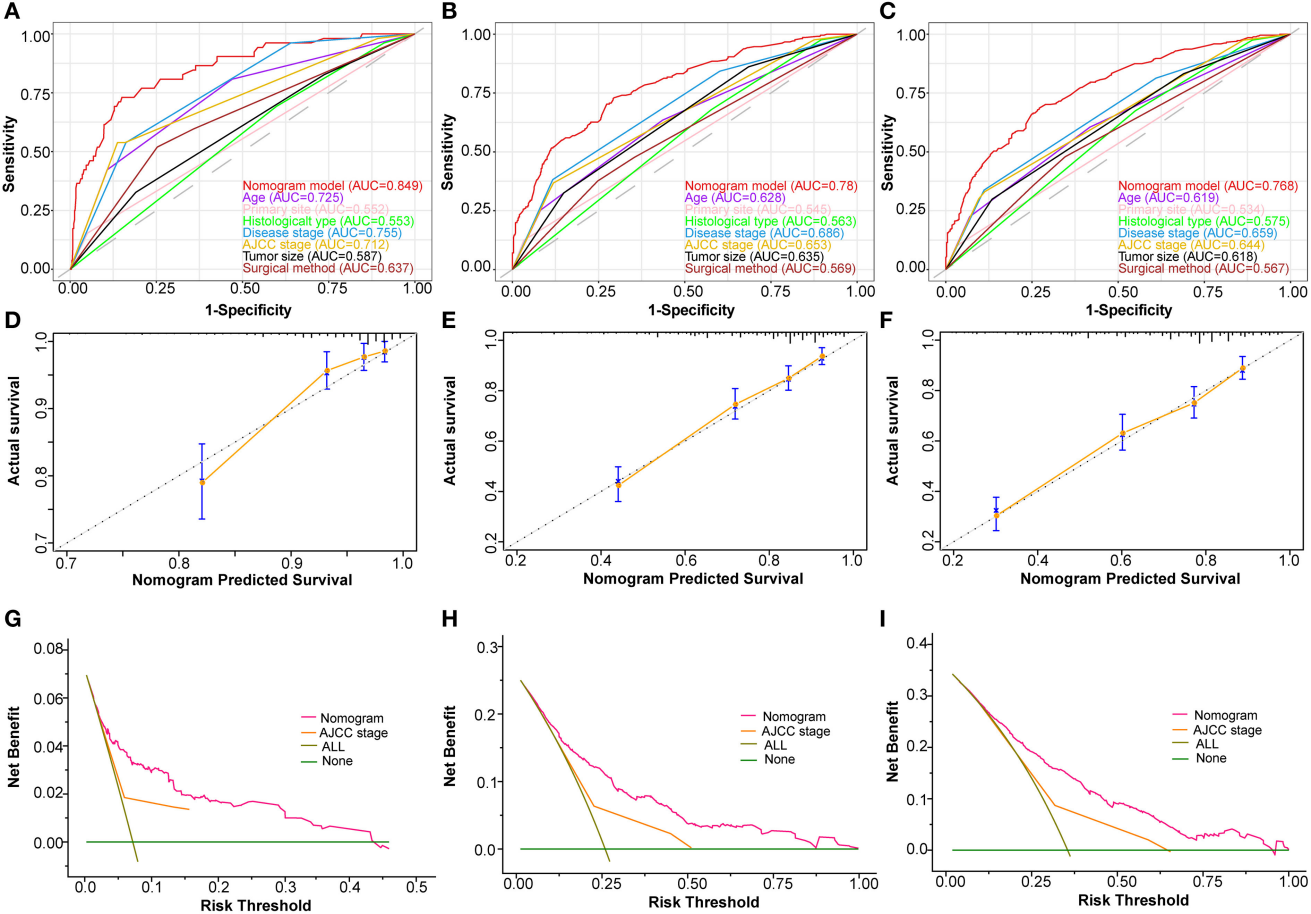
Validating the prognostic nomogram in the training cohort. Receiver operating characteristic curves (ROC) for 1 **(A)**, 3 **(B)**, and 5 **(C)** years in the training cohort, comparing the predictive ability between the nomogram and all independent factors, including age, primary site, histological type, disease stage, AJCC Stage, tumor size, and surgery. The calibration curves of the nomogram for 1 **(D)**, 2 **(E)**, and 3 **(F)** years in the training cohort. The decision curve analysis of the nomogram for 1 **(G)**, 2 **(H)**, and 3 **(I)** years in the training cohort, comparing the predictive ability of the nomogram with the AJCC stage.

**Figure 4:**
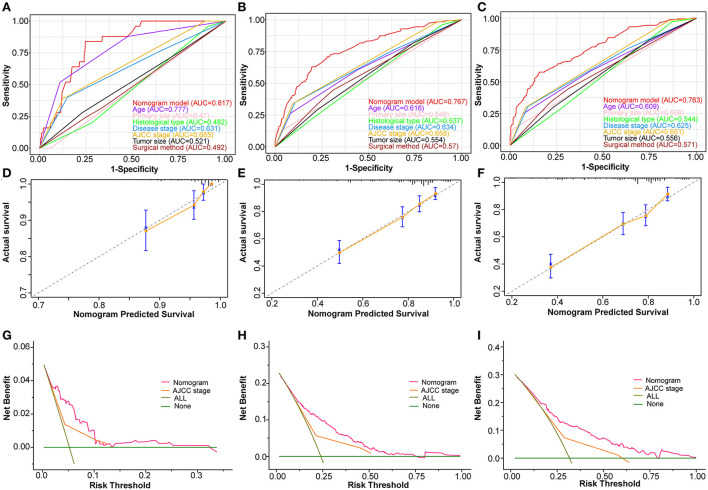
Validating the prognostic nomogram in the validation cohort. Receiver operating characteristic curves (ROC) for 1 **(A)**, 3 **(B)**, and 5 **(C)** years in the validation cohort, comparing the predictive ability between the nomogram and all independent factors, including age, primary site, histological type, disease stage, AJCC Stage, tumor size, and surgery. The calibration curves of the nomogram for 1 **(D)**, 2 **(E)**, and 3 **(F)** years in the validation cohort. The decision curve analysis of the nomogram for 1 **(G)**, 2 **(H)**, and 3 **(I)** years in the validation cohort, comparing the predictive ability of the nomogram with the AJCC stage.

Based on the abovementioned independent prognostic factors, we developed the first novel nomogram to predict OS after surgical resection in patients with primary osteosarcoma (Figure 2). Doctors and osteosarcoma patients could calculate a total point based on the scores of each independent prognostic variable and draw vertical lines between the “Total Points” and the axes of survival probability of 12-, 36-, and 60 months OS to estimate a patient's survival rate. As seen in Figure 2, this given patient's total point was 363 points, and the probability of survival of 1-, 3-, and 5 years was 92.86, 70.5, and 58.2%, respectively. Regarding the training set, the C-index of the nomogram was considerably higher than those of the AJCC stage [(HR: 0.741, 95% CI: 0.726–0.755) vs. (HR: 0.632, 95% CI: 0.619–0.645)]. In the validation set, the result was similar [(HR: 0.735, 95% CI: 0.718–0.753) vs. (HR: 0.635, 95% CI: 0.619–0.652)]. In the ROC curves of the training set, the 1-, 3-, and 5-year AUCs were considerably higher than those of the AJCC stage (0.849, 0.78, 0.768 vs. 0.712, 0.653, 0.644). In the validation set, the result was similar (0.817, 0.767, 0.763 vs.0.665, 0.658, 0.651). The C-index for both training and validation sets was more than 0.735 and the AUCs were more than 0.763, indicating that the nomogram had a better predictive ability than the AJCC stage (Figures 3A–C, 4A–C). The calibration curves revealed excellent agreement between the nomogram's predictions and the actual survival probabilities (Figures 3D–F, 4D–F). In DCA, the curves of the nomogram are above the AJCC-stage curves, indicating that our nomogram has more favorable clinical net benefits than the traditional AJCC stage (Figures 3G–I, 4G–I).

### Risk stratification system

**Figure 5:**
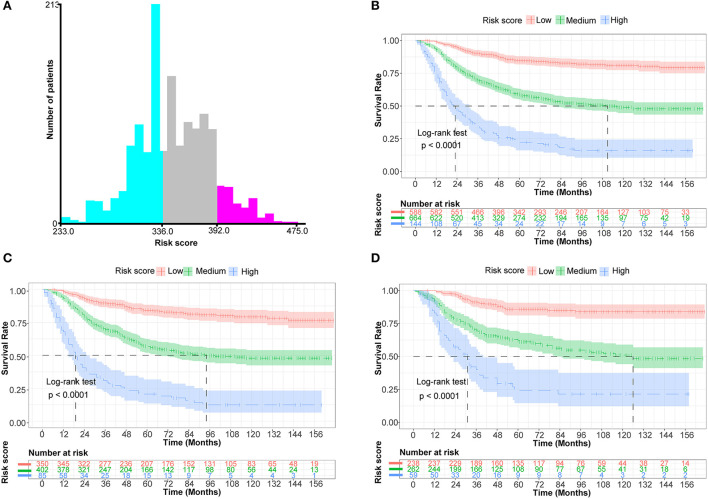
Establish a risk stratification system by determining the optimal cut-off point for risk scores. **(A)** Histogram of the distribution of patients based on the optimal cut-off point for risk scores (X-tile software). **(B)** Prognostic curves among distinct risk groups of all cohorts (the Kaplan-Meier survival analysis). **(C)** Prognostic curves among different risk groups of the training cohort. **(D)** Prognostic curves among different risk groups of the validation cohort.

X-tile program determined the optimal cut-off value for risk stratification based on the overall nomogram score of all patients (Figure 5A). All patients in this study were divided into three groups: low-risk (*N* = 588, 42.12%, scores <336), medium-risk (*N* = 664,47.56%, scores between 336 and 392), and high-risk (*N* = 144, 10.32%, scores >392). KM survival curves and log-rank tests demonstrated significant differences among the three risk groups (*p* < 0.001) in the whole cohort, the training set, and the validation set, indicating the validity of the nomogram-based risk stratification system (Figures 5B–D).

### Web-based nomogram

**Figure 6:**
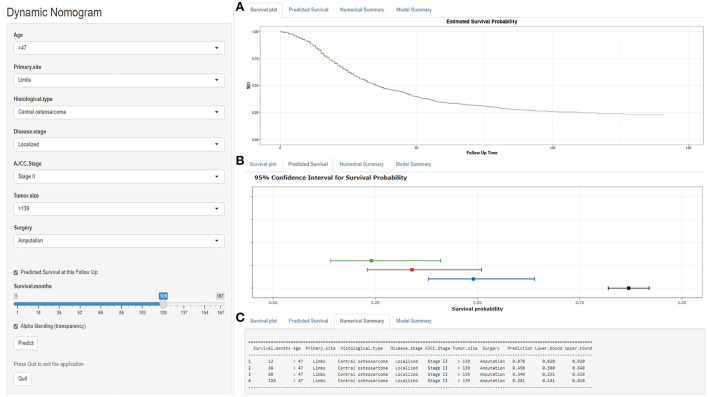
A web-based nomogram for predicting OS after surgical resection in patients with primary osteosarcoma. **(A)** The curve of the estimated probability of survival for this patient over time. **(B)** 95% confidence intervals of the 12-, 36-, 60-, and 120-month survival probabilities for this patient. **(C)** Numerical summary of the 12-, 36-, 60-, and 120-month survival probabilities for this patient. Due to a large number of visitors to the webpage, if the application cannot be used normally, please click “Quilt” or “Reload” in the lower-left corner to try again.

As seen in Figure 6, we developed a web-based nomogram for predicting OS after surgical resection in patients with primary osteosarcoma, allowing Doctors and patients to select common clinical variables to assess each postoperative patient's survival probability individually and visually. For example, we included a 50-year-old patient with a 150-mm-sized, localized stage-II central osteosarcoma of the limbs. After undergoing amputation, the estimated survival probabilities for this patient at 1-, 3-, 5-, and 10-year- were 87.0%(82–92%), 49.0%(38–64%), 34.0%(23.1–51%), and 24.1%(14.1–41%), respectively (https://gaobing2022.shinyapps.io/Nomogram_for_Postoperative_Osteosarcom/).

## Discussion

Before the 1970s, the surgical treatment of choice for osteosarcoma was amputation. Following the development of chemotherapy regimens, limb salvage surgery has allowed 70%-80% of osteosarcoma patients with a 5-year survival rate of 70% (32). Limb salvage provides aesthetic benefits, but it remains controversial whether it can completely replace amputation (6, 32). According to recent meta-analyses and systematic reviews, patients with limb salvage had better functional outcomes than amputation. However, selection bias may exist due to the relatively low incidence of osteosarcoma and the limited sample size (13, 33). The SEER database contains roughly 30% of the entire U.S. population, providing enough sample size for studies of rare malignancies and making statistical analysis more accessible (23). This study screened 1,396 osteosarcoma patients with complete follow-up data and treatment information on the SEER database.

Survival assessment during the perioperative period is crucial to predicting the prognosis of malignant osteosarcoma. Zhang et al. and Zhao et al. established nomograms for specific survival in osteosarcoma patients (20, 21). Wu et al. constructed a novel risk-score model based on osteosarcoma-related genes (22). Chen et al. built two nomograms to predict the risk probability and survival rate of distant metastasis in newly diagnosed osteosarcoma patients (11). These nomograms either did not incorporate the surgery-related variable or failed to distinguish between the impact of various surgical methods on survival. Prior surgery-related research for osteosarcoma has only identified survival risk factors after amputation or compared survival rates between amputation patients and limb-salvage patients (14–16, 34). Still, they have failed to differentiate between local and radical resection and have not presented reliable postoperative prognostic models. Nomograms have been proved to have superior predictive accuracy than the current AJCC stage in previous analyses of prognostic cancer models after surgical resection, such as gastric cancer, lung cancer, cholangiocarcinoma, and other malignancies (35–37). To the best of our knowledge, this was the first study to establish and validate a prognostic nomogram that was not inferior to the AJCC staging system to predict 1-,3- and 5-year OS for osteosarcoma patients after surgical resection. The current nomogram identified age, primary site, histological type, disease stage, AJCC stage, tumor size, and surgical method as independent prognostic factors for OS, which could be easily obtained from routine clinical data.

Age and tumor size have been widely reported as independent prognostic factors for osteosarcoma (14, 15, 38). The nomogram of OS and the KM survival curve indicated that the older the patient or the larger the tumor, the higher the nomogram score and the lower the prognostic survival rate (Figure 2, Supplementary Figures S1C,D). In terms of disease stage, non-metastatic stages (local and regional) confined to the periosteum or adjacent tissues have a better prognosis than distant stages (28). This may be because distant-stage osteosarcomas are mostly high-grade tumors that have spread to distant organs, with most metastases to the lung (60%-70%), followed by distal bone (20%-30%). The involvement of distant organs hinders the disease from responding to treatment and is the primary cause of death in osteosarcoma patients (1, 4). AJCC stage was determined to be an independent risk factor for postoperative osteosarcoma patients after univariate and multivariate analysis. Thus, we included them in the nomogram. The higher the stage, the worse the prognosis of the patient. We plotted ROC curves for the nomogram and all independent prognostic factors and presented the AUCs of all factors directly. Compared with the AJCC stage alone, the C-index and AUCs of the nomogram included in the AJCC stage were much higher, indicating that the nomogram was more accurate than the AJCC stage alone. DCA also demonstrated that the nomogram combined with the AJCC stage had a higher net clinical benefit, proved in both training and validation sets. The histological type markedly impacts the prognosis of patients with osteosarcoma. Patients with peripheral osteosarcoma had the best prognosis, which was consistent with a recent study published by Tian et al. (38). The classic central subtype is almost always a high-grade (grade III/IV) malignancy with a poor prognosis because it is poorly differentiated or undifferentiated and frequently spreads to distant organs. In contrast, most peripheral osteosarcomas are indolent low-grade (grade I/II) tumors, less prevalent but well-differentiated (4–6).

It's worthy of note that, unlike previous comparative analyses, we considered not only patients with osteosarcoma of the limbs but also the pelvis (15, 16). As seen by the nomogram and the multivariate analysis, the prognosis of pelvis osteosarcoma was substantially worse than that of limbs (HR > 1, *P* < 0.001) (Table 2), in line with prior reports (28, 39). Some scholars point out that surgical resection combined with salvage achieves optimal functional outcomes in the limbs (32). However, salvage surgery is rarely feasible in axial skeletal osteosarcoma, especially the pelvic. Pelvic tumors often cannot be radically removed due to the specificity of the surrounding accessory anatomical structures, and salvage surgery seldom yields satisfactory functional outcomes. Moreover, the reconstruction is also technically challenging, given the vast size of the pelvis. Amputation, therefore, remains an inescapable alternative in these cases (4, 6, 28).

Taking “amputation” as a reference, “radical resection” offered the most excellent prognosis. It was undoubtedly the best surgical method since it was an apparent protective factor in multivariate analysis (HR <1, *P* < 0.05) (Table 2) and stayed higher than other curves in KM survival analysis (Figure 1A). Tian et al. reported that local resection might be a relative protective factor for prognosis (38). But in this study, it was insignificant in the KM survival curve and the univariate COX regression analysis (HR < 1, *P* > 0.05). Therefore, in the nomogram, “amputation” and “local resection” had overlapping parts, which were difficult to discern considerably, and their prognoses were both worse than those of “radical resection.” This might be because the surgical lesions could be eliminated as much as feasible through radical resection to obtain a clear surgical margin (2, 7). But after local resection, there might still be residual tumor lesions with a chance of recurrence and an unfavorable prognosis (6). The worst course of option, however, would be amputation. Once osteosarcoma patients are forced to amputate their limbs, restoring desirable limb function and psychosocial outcomes are exceptionally challenging, even with prosthetic limbs (39, 40). As a result, orthopedic surgeons could recommend eligible patients with osteosarcoma choose radical resection as the optimal surgical method if the surgical indication is satisfied.

Osteosarcoma is generally considered a radiation-resistant tumor, and whether it should be treated with radiotherapy remains controversial (1, 2). The role of radiotherapy in osteosarcoma is far less defined than that of surgery and chemotherapy, and there is a lack of recognized standard treatment alternatives and clinical trial evidence. Given the unique anatomical structure of intralesional resection margins in the pelvis, skull, etc., osteosarcoma cannot be radically removed by surgery, and there will still be microscopic residual lesions (4, 6, 28). Palliative radiotherapy has been reported to help relieve metastatic pain and prolong survival in such cases (2, 40). In this study's multivariate analysis, radiotherapy was a relative risk factor for prognosis rather than a protective factor, but it was not statistically significant (HR > 1, *P* > 0.05) (Table 2). This is in line with what Tian et al. and Huang et al. reported. According to the nomogram presented by Zhang et al., patients who underwent radiotherapy still had a worse prognosis than those who did not. Therefore, more persuasive evidence-based medicine data and clinical trials are required to determine whether postoperative osteosarcoma patients should receive radiotherapy. Since the 1970s, Surgical resection combined with chemotherapy has become the cornerstone of treating patients with osteosarcoma (1, 2, 4, 40). However, the effect of chemotherapy on prognosis in this study was insignificant. Similar issues have arisen in earlier studies (15, 34, 38). This may be because almost all postoperative osteosarcoma patients received chemotherapy (Yes, 86.5%) (Table 1). The remaining patients (No/Unknown, 13.5%) were unable to confirm as not receiving due to incomplete chemotherapy information. Furthermore, the statistical validity of this study may also be limited by the absence of specific-chemotherapy regimens, degrees of response, and patient compliance in the SEER database.

In addition, as detailed in previous studies, the algorithms in X-tile enable quite reliable optimal cut-point analysis, determine the best cut-off value for continuous variables such as age and tumor size, or create survival-based risk stratification systems (24, 31). As a result, it has been frequently utilized in the survival analysis of malignancies such as breast cancer, cholangiocarcinoma, gastric carcinoma, osteosarcoma, and others (12, 20, 35, 37, 38, 41). In this study, a risk stratification system was constructed based on the nomogram scores of all patients. The three risk groups displayed substantial differences consistently in KM survival curves for the whole cohort, training set, and validation set, demonstrating the validity of the risk stratification system (Figure 5). Subsequently, we developed a web-based, user-friendly, dynamic nomogram that physicians and patients could access from any electronic device (Figure 6).

In recent years, Numerous incredible achievements have been witnessed in the clinical treatment and care of patients with osteosarcoma. New intelligent projects such as tailored 3D-printing technology and computer-aided navigation systems have achieved unprecedented development in osteosarcoma surgery (9, 10). Based on evidence-based medical data such as pathophysiology and medical imaging, the multidisciplinary medical center has jointly formulated novel expert-consensus guidelines for diagnosis, treatment, and follow-up (2). Despite this, the prognostic and survival improvement is still limited in osteosarcoma patients, with the 5-year survival rate constantly hovering at 70% (3, 4, 32). According to certain academics, individualized, intelligent medical services could offer fresh perspectives on enhancing the prognosis and survival of refractory diseases, including malignant tumors (17, 40, 42). During the SARS-CoV-2 outbreak, AI-based clinical predictive models have illustrated efficacy in anticipating epidemic trends and peaks, even facilitating the diagnosis and prognostic process of each patient with coronavirus disease (COVID-19) (43). This study developed a risk stratification system and a visual dynamic nomogram to individually and intuitively assess each patient's risk level and postoperative survival probability. This could be applied in clinical practice to support prospective treatment decisions. Doctors could tailor precise prognostic analysis for each osteosarcoma patient based on seven routine clinical variables such as age and tumor size and customize subsequent treatment and follow-up strategies.

It is worth noting that although the nomogram performs exceptionally well in terms of prediction, there are still some limitations. First, this study was designed as a retrospective analysis, so selection bias is unavoidable. Besides, judging the postoperative prognosis of osteosarcoma patients only based on survival/death is not enough. The SEER database lacks detailed information about chemotherapy regimen and response, surgical complications, functional recovery scores, etc. Moreover, there are very few databases with large samples and open access like the SEER database. Although our population-based prognostic nomogram proved to be effective following internal and external validation, multicenter patient data validation is still required to assess the clinical utility of the nomogram. The above requires further proof and refinement in the future.

## Conclusion

We highlighted that radical surgery was the first choice for patients with primary osteosarcoma since it provided the best survival prognosis. For the first time, we did establish and validate a novel nomogram that objectively predicts 1-, 3-, and 5-year overall survival in patients with primary osteosarcoma after surgical resection, with more accurate predictive performance and clinical utility than the AJCC stage. In addition, a web-based nomogram and risk stratification system could assist orthopedic surgeons in assessing survival prognosis, adjusting clinical decision-making strategies, and improving individualized survival probability.

## Data availability statement

The raw data supporting the conclusions of this article will be made available by the authors, without undue reservation.

## Author contributions

BG and FH formulated the study. BG and M-dW collected and analyzed the data and drafted the manuscript. YL and FH prepared the images. YL and FH proofread the manuscript. All authors approved the final version of the manuscript.

## Funding

This study was supported by the Natural Science Foundation of Jilin Province (No. 20210101296JC).

## Conflict of interest

The authors declare that the research was conducted in the absence of any commercial or financial relationships that could be construed as a potential conflict of interest.

## Publisher's note

All claims expressed in this article are solely those of the authors and do not necessarily represent those of their affiliated organizations, or those of the publisher, the editors and the reviewers. Any product that may be evaluated in this article, or claim that may be made by its manufacturer, is not guaranteed or endorsed by the publisher.

## Supplementary material

Supplementary Figure S1 Determining the optimal cut-off point for age and tumor size. **(A)** Histogram of the distribution of patients based on the optimal cut-off point for age (X-tile software). **(B)** Prognostic curves among distinct age subgroups (the Kaplan-Meier survival analysis). **(C)** Histogram of the distribution of patients based on the optimal cut-off point for tumor size. **(D)** Prognostic curves among distinct tumor size subgroups.
