# Supplementary figures and images for: Risk stratification system and web-based nomogram constructed for predicting the overall survival of primary osteosarcoma patients after surgical resection
Source: Front Public Health. 2022 Aug 5;10:949500. doi: 10.3389/fpubh.2022.949500 (PMC9389295; doi:10.3389/fpubh.2022.949500)

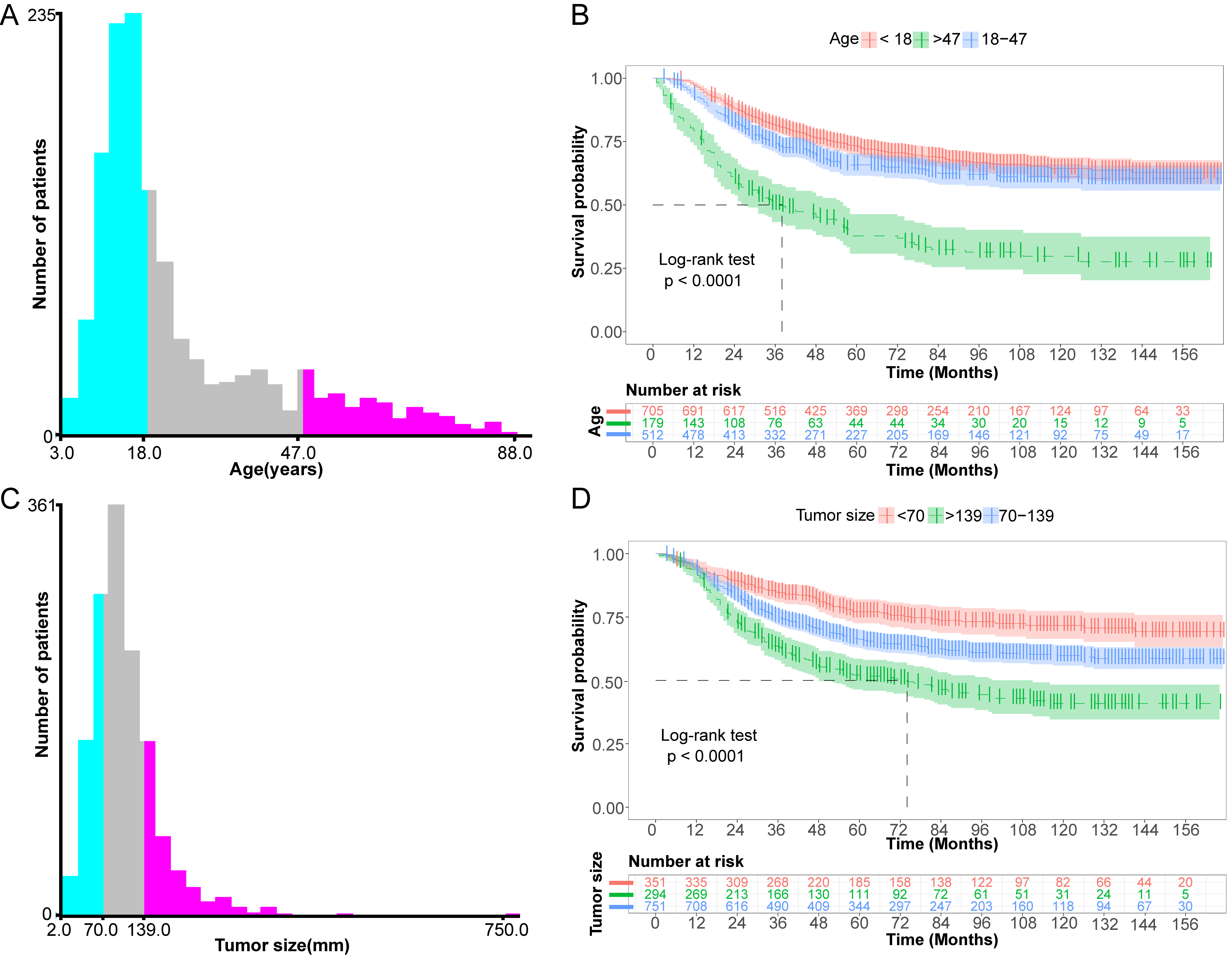

Supplement: Supplementary Figure S1 — Determining the optimal cut-off point for age and tumor size. (A) Histogram of the distribution of patients based on the optimal cut-off point for age (X-tile software). (B) Prognostic curves among distinct age subgroups (the Kaplan-Meier survival analysis). (C) Histogram of the distribution of patients based on the optimal cut-off point for tumor size. (D) Prognostic curves among distinct tumor size subgroups. [file Image_1.TIF]
